# Supplementary figures and images for: Sixty years of change in avian communities of the Pacific Northwest
Source: PeerJ. 2015 Aug 4;3:e1152. doi: 10.7717/peerj.1152 (PMC4558065; doi:10.7717/peerj.1152)

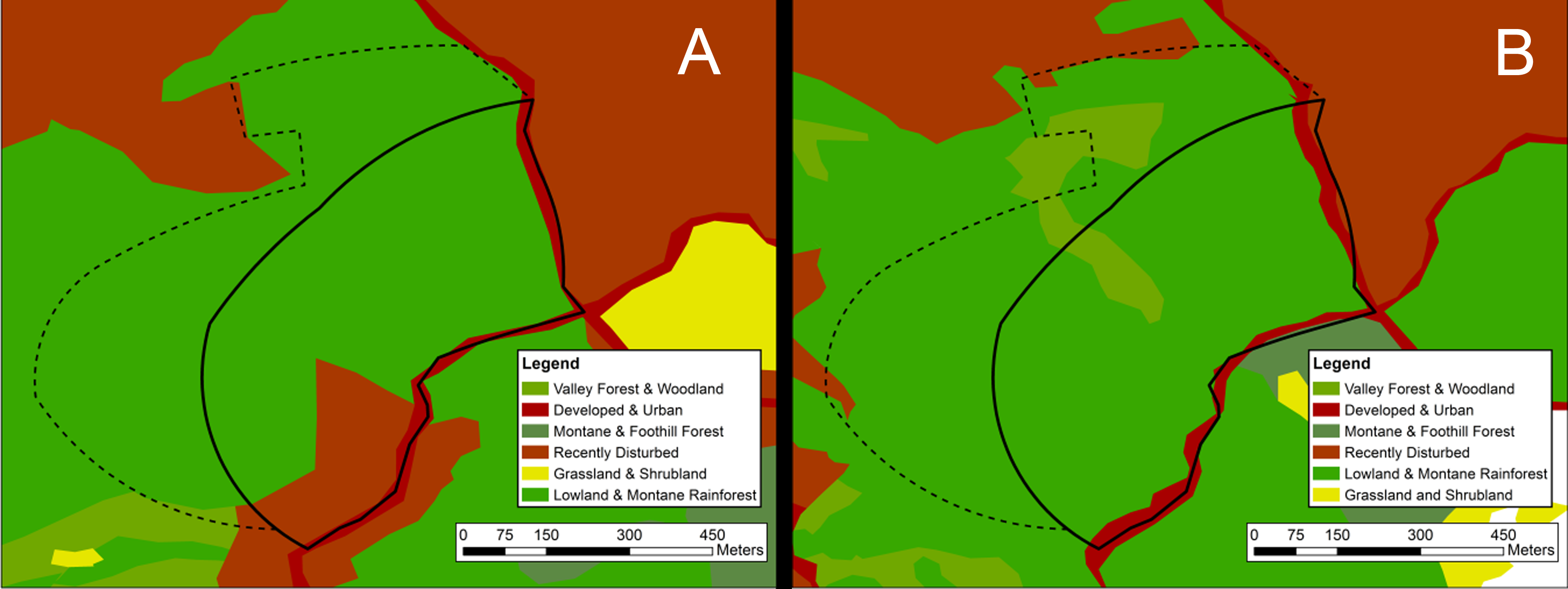

Supplement: Figure S1 — Classifications based on 1956 aerial photographs (A) and 2012 satellite imagery (B). The boundary of the 14-hectare survey site is marked in black. The black dashed line indicates a “likely area” to which the historic site boundaries may have extended. Vegetation system categories derived from NVC levels in the National Gap Analysis Program (GAP) (US Geological Survey, 2012). [file peerj-03-1152-s001.png]

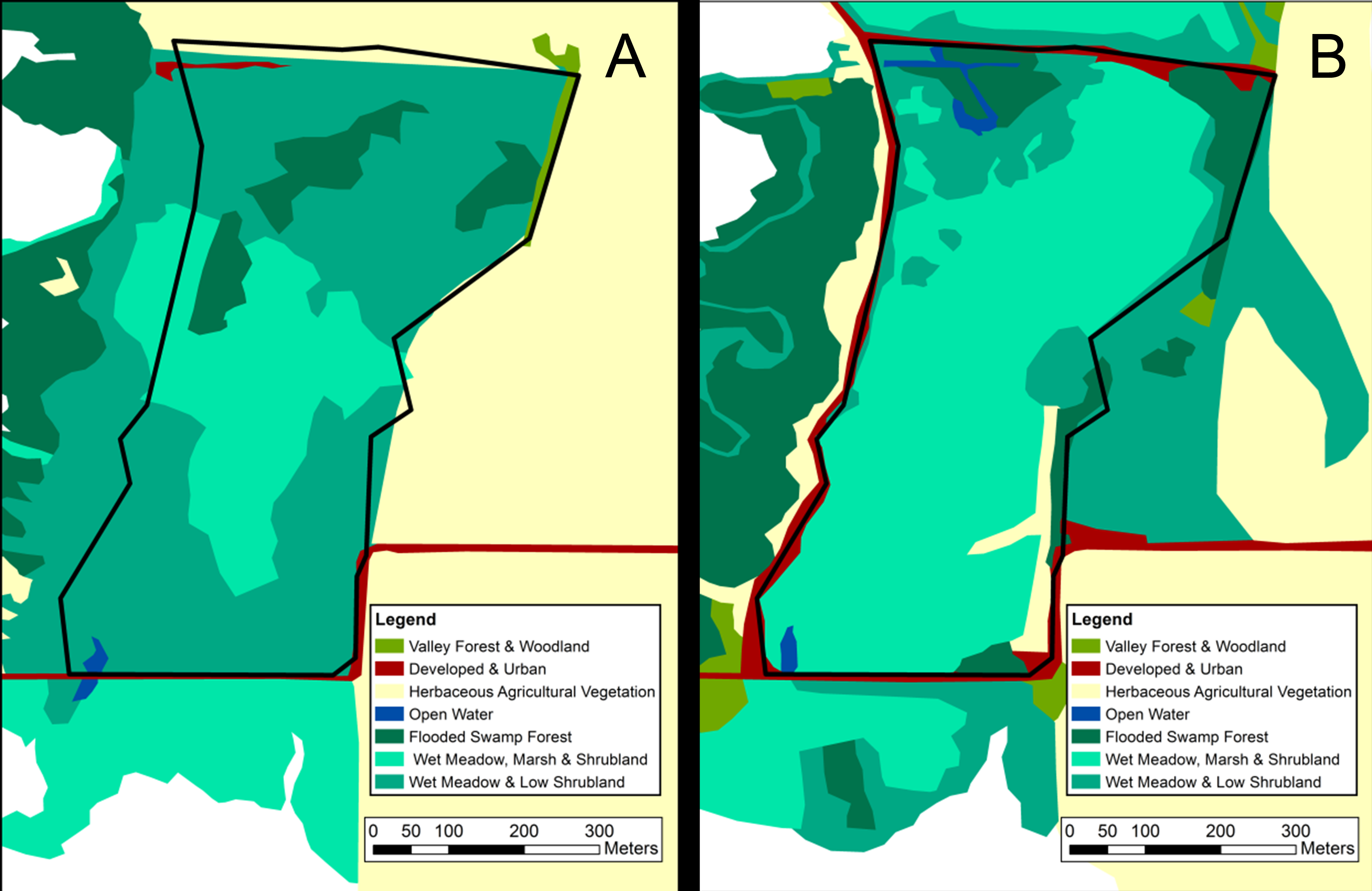

Supplement: Figure S2 — Classifications based on 1956 aerial photographs (A) and 2012 satellite imagery (B). The boundary of the 20-hectare survey site is marked in black. Vegetation system categories derived from NVC levels in the National Gap Analysis Program (GAP) (US Geological Survey, 2012). [file peerj-03-1152-s002.png]

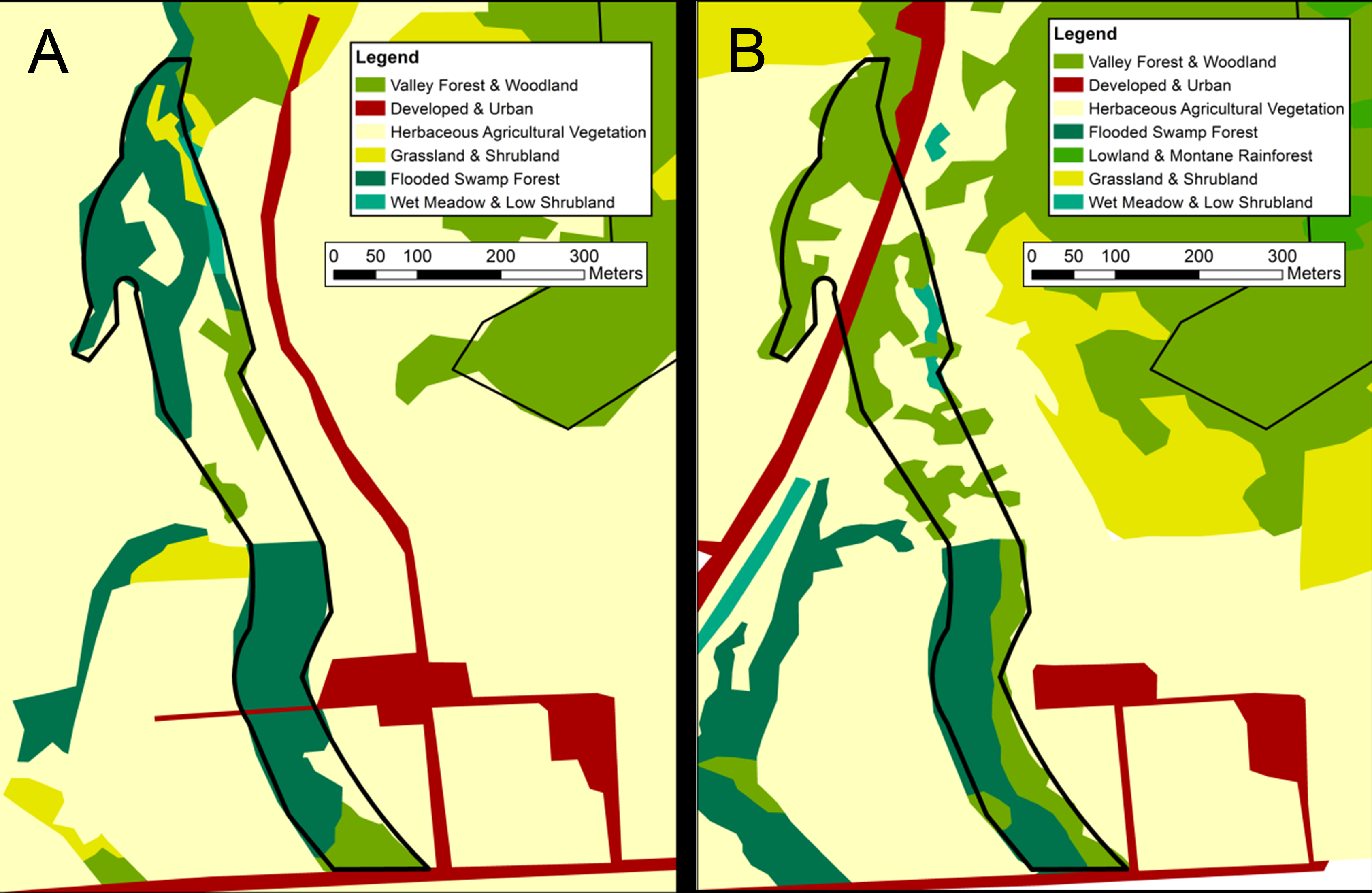

Supplement: Figure S3 — Classifications based on 1956 aerial photographs (A) and 2012 satellite imagery (B). The boundary of the 8-hectare survey site is marked in black. Vegetation system categories derived from NVC levels in the National Gap Analysis Program (GAP) (US Geological Survey, 2012). [file peerj-03-1152-s003.png]

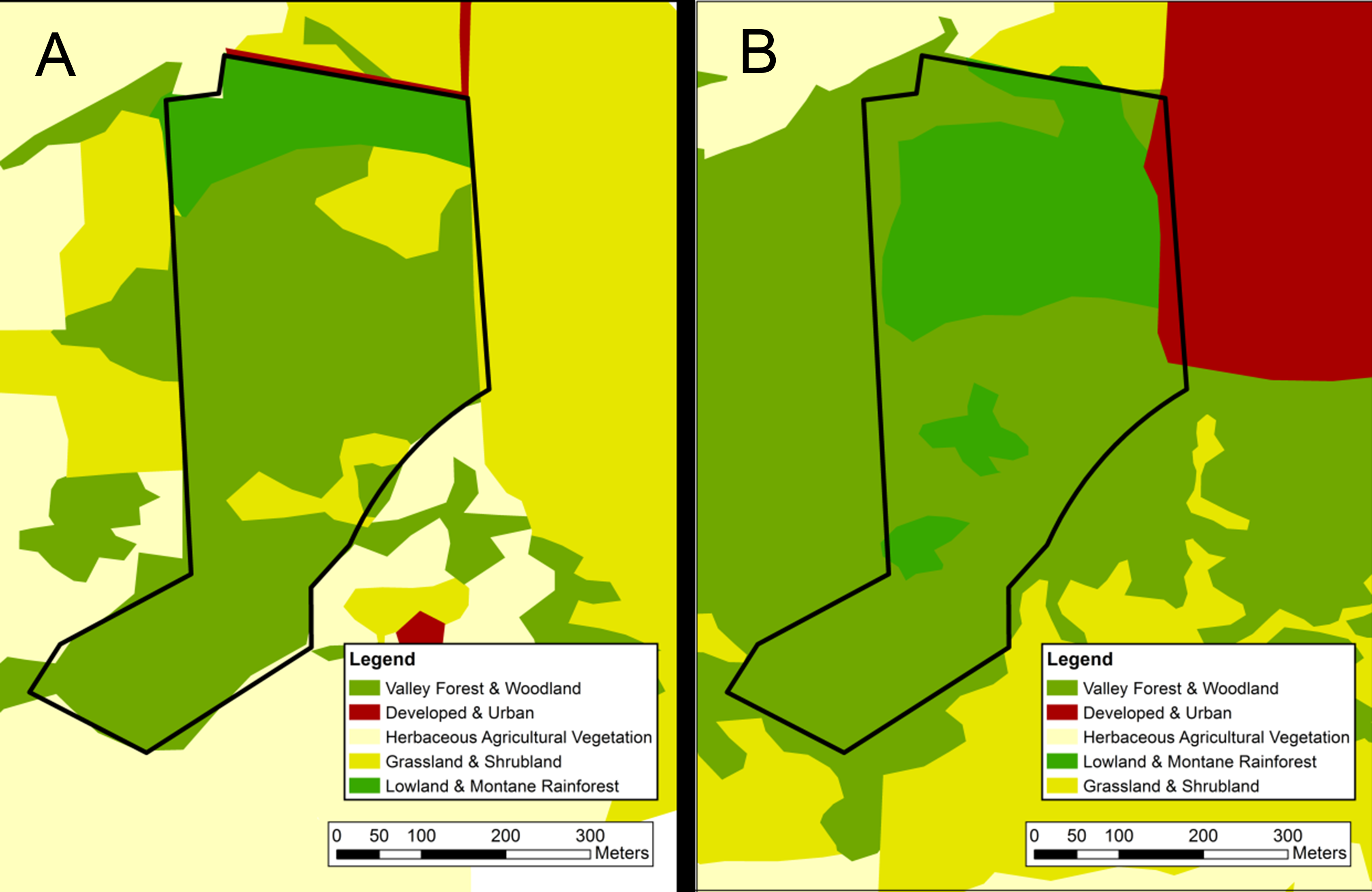

Supplement: Figure S4 — Classifications based on 1956 aerial photographs (A) and 2012 satellite imagery (B). The boundary of the 14-hectare survey site is marked in black. The black dashed line indicates a “likely area” to which the historic site boundaries may have extended. Vegetation system categories derived from NVC levels in the National Gap Analysis Program (GAP) (US Geological Survey, 2012). [file peerj-03-1152-s004.png]

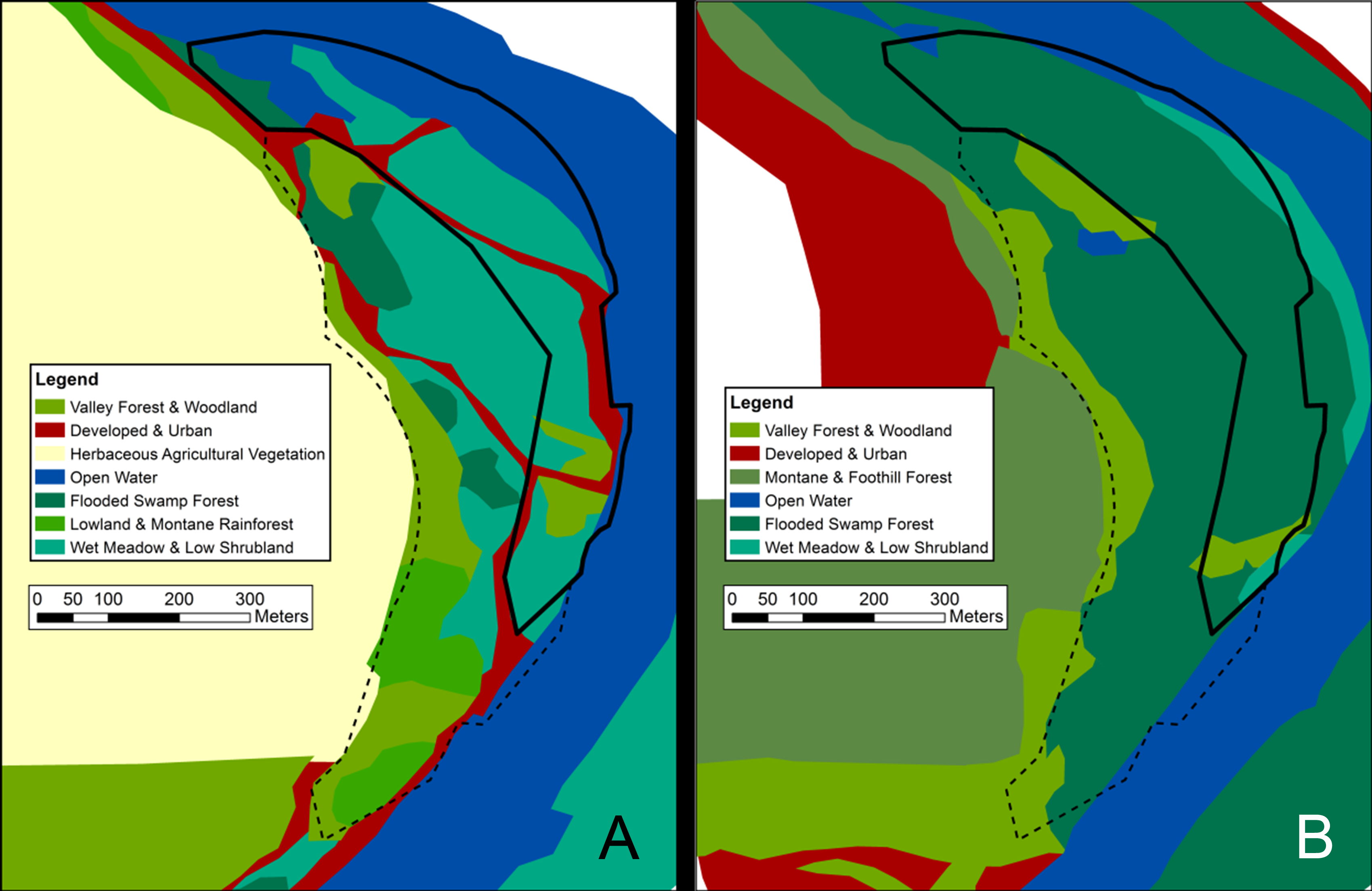

Supplement: Figure S5 — Classifications based on 1956 aerial photographs (A) and 2012 satellite imagery (B). The boundary of the 10-hectare survey site is marked in black. Vegetation system categories derived from NVC levels in the National Gap Analysis Program (GAP) (US Geological Survey, 2012). [file peerj-03-1152-s005.png]
