# Supplementary material for: Sixty years of change in avian communities of the Pacific Northwest
Source: PeerJ. 2015 Aug 4;3:e1152. doi: 10.7717/peerj.1152 (PMC4558065; doi:10.7717/peerj.1152)
Supplement: Table S1 — Species sorted in alphabetic order by abundance category. “Proposed explanation” represents the authors’ best justification as to why those species were not detected. Abundance categories based on average number of individuals detected during each survey across all sites. Breeding Bird Survey (BBS) population trends for Oregon obtained from Sauer et al. (2014). Negative numbers indicate declining trends. [file peerj-03-1152-s006.docx]

| Abundance Category | Species | Proposed Explanation | BBS Trend (OR) |
| --- | --- | --- | --- |
| Common | California Quail | Changes in surrounding habitat and succession. Seen only at Willamette site. | -0.2 |
|  | Chipping Sparrow | Regionally declining, vegetation succession. | -2.8 |
|  | Golden-crowned Kinglet | Regionally declining, changes to understory. | -3.4 |
|  | House Sparrow | Changes in surrounding habitat, vegetation succession. Seen only at Willamette site. | -1.9 |
|  | Nashville Warbler | Vegetation succession, changes to understory. Seen only at Oak woodland site. | -0.8 |
|  | Northern Rough-winged Swallow | Regionally declining, interannual changes in emergent insect levels. | -3.1 |
|  | Western Tanager | Sampling differences. We detected, but not visually. | 0.3 |
| Uncommon | Brewer's Blackbird | Regionally declining. We detected, but not during 2 hour survey period. | -2.7 |
|  | Band-tailed Pigeon | Sampling differences. We detected, but not visually. | -0.3 |
|  | Black-throated Gray Warbler | Sampling differences. We detected, but not visually. | -2.9 |
|  | Cassin's Vireo | Vegetation succession at Mixed Deciduous site. All other sites: We detected, but not during 2 hour survey period. | -0.9 |
|  | Common Nighthawk | Regionally declining, changes to surrounding habitat. | -1.0 |
|  | Hairy Woodpecker | Sampling differences. We detected, but not visually. | 0.3 |
|  | House Wren | Vegetation succession, closure of canopy, and changes to understory. Regionally declining. Seen only at Oak woodland site. | -3.4 |
|  | MacGillivray's Warbler | Widespread regional decline. Changes to understory. | -2.0 |
|  | Northern Bobwhite | Introduced species, regionally declining. Changes in surrounding habitat. | -6.6 |
|  | Olive-sided Flycatcher | Regionally declining, changes to canopy layer, vegetation succession | -3.1 |
|  | Ruffed Grouse | Changes to understory layer, closure of canopy at Coniferous site. | 1.3 |
|  | Townsend's Warbler | Suspected late migrants. | -0.6 |
|  | White-crowned Sparrow | Changes to understory layer, vegetation growth, and closure of canopy at Coniferous site. | -3.7 |
|  | Western Bluebird | Changes to understory layer, vegetation growth, and closure of canopy at Coniferous site. | 1.3 |
| Rare | American Bittern | Sampling differences. We detected, but not visually. | -3.0 |
|  | American Kestrel | Regionally declining, changes in surrounding habitat. | -1.4 |
|  | Common Merganser | Sampling differences. We detected, but not visually. | -0.9 |
|  | Fox Sparrow | Species likely misidentified (probably SOSP). | -0.2 |
|  | Gray Jay | We detected, but not during 2 hour survey period. | 1.1 |
|  | Pine Siskin | Regionally declining. Interannual differences in resource availibility and resulting distribution shifts. | -5.2 |
|  | Pileated Woodpecker | We detected, but not during 2 hour survey period. | 1.5 |
|  | Ring-necked Pheasant | Introduced species, regionally declining. Changes in surrounding habitat. | -3.2 |
|  | Sora | We detected, but not visually and not during 2 hour survey period. Eddy made efforts to flush secretive birds. | -0.8 |
|  | Unid. Empidonax Flycatcher | We was able to identify all Empidonax sp. to the species level. | -- |
|  | Warbling Vireo | Sampling differences. We detected, but not visually. | 0.0 |
|  | Western Meadowlark | Urban development, vegetation succession, and changes to surrounding habitat. | -1.3 |
|  | Wilson's Snipe | Sampling differences. We detected, but not visually. | -1.4 |
|  | Yellow-breasted Chat | Area no longer grazed, succession and changes to surrounding vegetation. Seen only at Marsh site. | -0.9 |
