# Supplementary material for: Sixty years of change in avian communities of the Pacific Northwest
Source: PeerJ. 2015 Aug 4;3:e1152. doi: 10.7717/peerj.1152 (PMC4558065; doi:10.7717/peerj.1152)
Supplement: Table S2 — Species sorted in alphabetic order by abundance category. “Proposed explanation” represents the authors’ best justification as to why those species were not detected. Abundance categories based on average number of individuals detected during each survey across all sites. Breeding Bird Survey (BBS) population trends for Oregon obtained from Sauer et al. (2014). Negative numbers indicate declining trends. [file peerj-03-1152-s007.docx]

| Abundance Category | Species | Proposed Explanation | BBS Trend (OR) |
| --- | --- | --- | --- |
| Common | European Starling | Regional range expansion. | -0.9 |
|  | Unknown Duck | Eddy was able to identify all ducks to species level, we were not. | -- |
|  | Wilson's Warbler | Unknown. Likely vegetation succession and changes in understory density/composition. | -1.5 |
| Uncommon | Brown-headed Cowbird | Regional range expansion. | -1.8 |
|  | Cinnamon Teal | Area no longer grazed, increase in standing water at Marsh site. Only seen early in season while open standing water was available. | -4.8 |
|  | Evening Grosbeak | Interannual differences in resource availability and resulting distribution shifts. Eddy unlikely to visually detect. | -5.3 |
|  | House Finch | Regional range expansion. Eddy described within city limits but did not detect during surveys. | -1.4 |
|  | Pied-billed Grebe | Area no longer grazed, increase in standing water at Marsh site. | 1.2 |
|  | Spotted Sandpiper | Changes to shoreline composition along Willamette River. Increase in gravel shores and beds. | -1.5 |
| Rare | Acorn Woodpecker | Regionally increasing. Maturation of canopy at Oak woodland site. | 3.0 |
|  | Anna's Hummingbird | Regional range expansion. | 12.3 |
|  | Bald Eagle | Regionally increasing, range expansion. | 5.8 |
|  | Black Phoebe | Regional range expansion. | 4.3 |
|  | Canada Goose | Regionally increasing. Increase in nests along Willamette River. | 4.9 |
|  | Common Raven | Sampling differences, Eddy unlikely to have visually detected. Regionally increasing. | 2.2 |
|  | Eurasian Collared-dove | Regional range expansion. | 143.6 |
|  | Great Egret | Regional range expansion. Area no longer grazed, increase in standing water at Marsh site. | -0.2 |
|  | Greater Yellowlegs | Area no longer grazed, increase in standing water at Marsh site. Only seen early in season while open standing water was available. | -- |
|  | Green-winged Teal | Area no longer grazed, increase in standing water at Marsh site. Only seen early in season while open standing water was available. | -0.2 |
|  | Hammond's Flycatcher | Eddy did not identify Empidonax flycatchers to the species level. | 1.1 |
|  | Osprey | Regionally increasing. Sampling differences, Eddy unlikely to have visually detected. | 5.0 |
|  | Pacific-slope Flycatcher | Eddy did not identify Empidonax flycatchers to the species level. Eddy unlikely to have visually detected. | -2.7 |
|  | Savannah Sparrow | Flushed from adjacent unmowed grass field. Surrounding habitat no longer grazed. Seen only at Marsh site. | -2.3 |
|  | Sharp-shinned Hawk | Regionally increasing. Sampling differences, Eddy unlikely to have visually detected. | 3.8 |
|  | Unknown Hummingbird | Multiple hummingbird species not present in 1952. Eddy was able to identify all hummingbirds as Rufous. | -- |
|  | Virginia Rail | Regionally increasing. Sampling differences, Eddy unlikely to have visually detected. | 2.0 |
|  | Willow Flycatcher | Eddy did not identify Empidonax flycatchers to the species level. | -6.0 |
|  | Yellow-headed Blackbird | Local range expansion from nearby populations. | -2.2 |
