# Supplementary material for: Sixty years of change in avian communities of the Pacific Northwest
Source: PeerJ. 2015 Aug 4;3:e1152. doi: 10.7717/peerj.1152 (PMC4558065; doi:10.7717/peerj.1152)
Supplement: Table S3 — Species sorted in taxonomic order by trend category. Trends were classified based on the size of the shift in abundance categories between years. Species classified as “strongly” increasing or decreasing shifted more than two categories up or down, respectively. For species classified as “No Change”, (A) indicates species were generally abundant both years, while (R) indicates species were generally rare both years. ∗ denotes species that were not visually detected. [file peerj-03-1152-s008.docx]

| Population Trend | Species | Scientific Name |
| --- | --- | --- |
| No Change (A) | Downy Woodpecker | *Picoides pubescens* |
|  | Steller's Jay | *Cyanocitta stelleri* |
|  | American Crow | *Corvus brachyrhynchos* |
|  | Cliff Swallow | *Petrochelidon pyrrhonota* |
|  | Black-capped Chickadee | *Poecile atricapillus* |
|  | Bushtit | *Psaltriparus minimus* |
|  | Bewick's Wren | *Thryomanes bewickii* |
|  | American Robin | *Turdus migratorius* |
|  | Yellow Warbler | *Setophaga petechia* |
|  | Spotted Towhee | *Pipilo maculatus* |
|  | Song Sparrow | *Melospiza melodia* |
|  | Lazuli Bunting | *Passerina amoena* |
|  | Red-winged Blackbird | *Agelaius phoeniceus* |
| No Change (R) | Killdeer | *Charadrius vociferus* |
|  | Hairy Woodpecker | *Picoides villosus* |
|  | Pileated Woodpecker | *Dryocopus pileatus* |
|  | Townsend's Warbler | *Setophaga townsendi* |
| Increasing | Mallard | *Anas platyrhynchos* |
|  | Cinnamon Teal | *Anas cyanoptera* |
|  | Common Merganser * | *Mergus merganser* |
|  | Pied-billed Grebe | *Podilymbus podiceps* |
|  | Great Blue Heron | *Ardea herodias* |
|  | Great Egret | *Ardea alba* |
|  | Osprey * | *Pandion haliaetus* |
|  | Bald Eagle | *Haliaeetus leucocephalus* |
|  | Cooper's Hawk * | *Accipiter cooperii* |
|  | Red-tailed Hawk | *Buteo jamaicensis* |
|  | Sora * | *Porzana carolina* |
|  | Spotted Sandpiper | *Actitis macularius* |
|  | Mourning Dove | *Zenaida macroura* |
|  | Great Horned Owl | *Bubo virginianus* |
|  | Northern Pygmy-owl * | *Glaucidium gnoma* |
|  | Anna's Hummingbird | *Calypte anna* |
|  | Rufous Hummingbird | *Selasphorus rufus* |
|  | Belted Kingfisher | *Megaceryle alcyon* |
|  | Acorn Woodpecker | *Melanerpes formicivorus* |
|  | Red-breasted Sapsucker | *Sphyrapicus ruber* |
|  | Northern Flicker | *Colaptes auratus* |
|  | Western Wood-pewee | *Contopus sordidulus* |
|  | Willow Flycatcher * | *Empidonax traillii* |
|  | Hammond's Flycatcher * | *Empidonax hammondii* |
|  | Black Phoebe | *Sayornis nigricans* |
|  | Warbling Vireo | *Vireo gilvus* |
|  | Western Scrub-jay | *Aphelocoma californica* |
|  | Common Raven | *Corvus corax* |
|  | House Wren * | *Troglodytes aedon* |
|  | Marsh Wren | *Cistothorus palustris* |
|  | Swainson's Thrush | *Catharus ustulatus* |
|  | Cedar Waxwing | *Bombycilla cedrorum* |
|  | Orange-crowned Warbler | *Oreothlypis celata* |
|  | Common Yellowthroat | *Geothlypis trichas* |
|  | Dark-eyed Junco | *Junco hyemalis* |
|  | Yellow-headed Blackbird * | *Xanthocephalus xanthocephalus* |
|  | Purple Finch | *Haemorhous purpureus* |
|  | Lesser Goldfinch | *Spinus psaltria* |
|  | American Goldfinch | *Spinus tristis* |
| Decreasing | California Quail | *Callipepla californica* |
|  | Northern Bobwhite | *Colinus virginianus* |
|  | Ring-necked Pheasant | *Phasianus colchicus* |
|  | Ruffed Grouse | *Bonasa umbellus* |
|  | Band-tailed Pigeon | *Patagioenas fasciata* |
|  | American Kestrel | *Falco sparverius* |
|  | Olive-sided Flycatcher | *Contopus cooperi* |
|  | Cassin's Vireo | *Vireo cassinii* |
|  | Gray Jay | *Perisoreus canadensis* |
|  | Violet-green Swallow | *Tachycineta thalassina* |
|  | Barn Swallow | *Hirundo rustica* |
|  | White-breasted Nuthatch | *Sitta carolinensis* |
|  | Golden-crowned Kinglet | *Regulus satrapa* |
|  | Western Bluebird | *Sialia mexicana* |
|  | MacGillivray's Warbler | *Geothlypis tolmiei* |
|  | Black-throated Gray Warbler | *Setophaga nigrescens* |
|  | Yellow-breasted Chat | *Icteria virens* |
|  | White-crowned Sparrow | *Zonotrichia leucophrys* |
|  | Western Tanager | *Piranga ludoviciana* |
|  | Western Meadowlark | *Sturnella neglecta* |
|  | Brewer's Blackbird | *Euphagus cyanocephalus* |
|  | Pine Siskin | *Spinus pinus* |
| Strongly Increasing | Wood Duck | *Aix sponsa* |
|  | Virginia Rail | *Rallus limicola* |
|  | Pacific-slope Flycatcher | *Empidonax difficilis* |
|  | Tree Swallow | *Tachycineta bicolor* |
|  | Chestnut-backed Chickadee | *Poecile rufescens* |
|  | Red-breasted Nuthatch | *Sitta canadensis* |
|  | Brown Creeper | *Certhia americana* |
|  | Pacific Wren | *Troglodytes pacificus* |
|  | European Starling | *Sturnus vulgaris* |
|  | Hermit Warbler * | *Setophaga occidentalis* |
|  | Wilson's Warbler | *Cardellina pusilla* |
|  | Black-headed Grosbeak | *Pheucticus melanocephalus* |
|  | Brown-headed Cowbird | *Molothrus ater* |
|  | House Finch | *Haemorhous mexicanus* |
|  | Red Crossbill | *Loxia curvirostra* |
|  | Evening Grosbeak | *Coccothraustes vespertinus* |
| Strongly Decreasing | Turkey Vulture | *Cathartes aura* |
|  | Northern Rough-winged Swallow | *Stelgidopteryx serripennis* |
|  | Nashville Warbler | *Oreothlypis ruficapilla* |
|  | Chipping Sparrow | *Spizella passerina* |
|  | House Sparrow | *Passer domesticus* |
