# Supplementary material for: Sixty years of change in avian communities of the Pacific Northwest
Source: PeerJ. 2015 Aug 4;3:e1152. doi: 10.7717/peerj.1152 (PMC4558065; doi:10.7717/peerj.1152)
Supplement: Appendix S1 [file peerj-03-1152-s009.docx]

**Evaluating the effect of site placement through the use of “likely areas”**

To confirm site location did not affect survey results at sites with uncertain placement, we extended the 200m grid established within sites into the designated “likely areas”. We conducted five 5-minute stationary point counts at each grid intersection within both sites and “likely areas”. We used Student’s t-tests to evaluate the null hypothesis of no difference in mean Shannon-Wiener diversity across all counts within the designated site compared to diversity in adjacent “likely areas”. Using diversity, rather than simple abundances, also accounts for the evenness of abundances among species within the surveyed communities. There was no evidence that placing the site in the selected area rather than the adjacent region resulted in a different avian community at either site for which placement was uncertain (p > 0.5 for both sites). For additional details, please see: Curtis, J. 2015. 60 years of avian community change in the Willamette Valley, Oregon. Master’s Thesis. Corvallis: Oregon State University.
